# Exposure to Anthropogenic Areas May Influence Colonization by Zoonotic Microorganisms in Scavenging Birds

**DOI:** 10.3390/ijerph18105231

**Published:** 2021-05-14

**Authors:** Guillermo María Wiemeyer, Pablo Ignacio Plaza, Carla Paola Bustos, Alejandra Jimena Muñoz, Sergio Agustín Lambertucci

**Affiliations:** 1 Grupo de Investigaciones en Biología de la Conservación, INIBIOMA, CONICET-Universidad Nacional del Comahue, Quintral, San Carlos de Bariloche 1250 (R8400FRF), Argentina; plazapablo22@gmail.com (P.I.P.); slambertucci@comahue-conicet.gob.ar (S.A.L.); 2 The Peregrine Fund, 5668 West Flying Hawk Lane, Boise, ID 83709, USA; 3 Buenos Aires Zoo, República de la India 3000, CABA, Ciudad Autónoma de Buenos Aires CP1425, Argentina; 4 Hospital Escuela, Facultad de Ciencias Veterinarias, Universidad de Buenos Aires, Av. Chorroarín 280, Ciudad Autónoma de Buenos Aires CP1427, Argentina; 5 Cátedra de Enfermedades Infecciosas, Laboratorio Escuela Enfermedades Infecciosas, Facultad de Ciencias Veterinarias, Universidad de Buenos Aires, Av. Chorroarín 280, Ciudad Autónoma de Buenos Aires CP1427, Argentina; carlabustos@fvet.uba.ar (C.P.B.); amunoz@fvet.uba.ar (A.J.M.)

**Keywords:** Andean condor, anthropized environment, bacteria, *Salmonella*, vulture, zoonotic pathogens

## Abstract

Wild bird species have commonly been implicated as potential vectors of pathogens to other species, humans included. However, the habitat where birds live could influence the probability to acquire these pathogens. Here, we evaluated if the characteristics of the environment used by obligate scavenging birds (vultures) influence their colonization by zoonotic pathogens. For this, we particularly focused on *Salmonella* spp., a zoonotic pathogen commonly present in bird species. The occurrence of this bacteria was evaluated in free ranging Andean condors (*Vultur gryphus*) using natural environments from Argentina and compared with those obtained from condors under human care. In addition, we compared our results with those reported for other wild vultures using natural and anthropized environments at a global scale. We did not find *Salmonella* spp. in samples of wild condors. Captive condor samples presented *Salmonella* spp. with an occurrence of 2.8%, and one isolate of Meticilin Resistant *Staphylococcus aureus*, among other potential pathogenic microorganisms. Moreover, some species of free ranging vultures from diverse geographical areas using anthropized environments tend to present higher occurrences of *Salmonella* spp. These results highlight the importance of pristine ecosystems to protect vultures’ health toward pathogenic microorganisms that can produce disease in these birds, but also in other species. We call for more studies evaluating differences in occurrence of zoonotic pathogens in vultures according to the quality of the environment they use. Even when vultures have not been implicated in zoonotic pathogen spread, our results add information to evaluate potential events of pathogen spillover between vultures and from these birds to other species.

## 1. Introduction

In the past few decades, emerging infectious diseases originated from wildlife species have been considered an important global threat to human health [1,2]. Several pathogens (e.g., Hendra virus disease, or Hantavirus pulmonary syndrome, among others) have emerged from different wildlife species affecting their populations but also producing, in some cases, severe disease outbreaks in humans [1]. In fact, the pandemic event emerged in 2019, with millions of people being infected and many dying because of SARS-CoV-2 virus, was originated as a spillover probably from bats [3,4]. Understanding the diverse factors involved in pathogen and infectious disease emergence, under the one health perspective, it is key to maintain the ecosystem function in order to preserve human health. 

The emergence of pathogens is mainly driven by environmental, socio cultural, and ecological factors [2,5]. Diverse pathogens have emerged as a consequence of land use modifications or changes (e.g., changes produced by urbanization, livestock production, or deforestation among others), which create the necessary conditions that facilitate pathogen emergence, survival, and spread [5]. Currently land use changes produced by livestock production, deforestation, urbanization, or agriculture are responsible for important transformations of natural ecosystems, causing extensive environmental damage, which, in turn, contributes to the emergence of pathogens [6,7]. In fact, close contact between livestock, wildlife and humans associated with sites impacted by anthropic activities could favor pathogen spillover between this interface with consequences difficult to predict on human health [8].

Wild birds have been commonly implicated as potential vectors of pathogens to other species, humans included [9]. It is interesting to note that the habitat where birds live, forage, rest, and nest could influence the risk to acquire pathogens potentially dangerous for them but also for other species [9,10]. For instance, birds foraging close to intensive livestock productions show a higher occurrence of enteric pathogens or resistant bacteria compared to birds foraging far away these sites [10,11,12]. Similarly, birds foraging in anthropic sites, such as rubbish dumps or sewage, have more frequently been colonized by different zoonotic pathogens, such as *Salmonella* spp. [9,13]. Therefore, the characteristics of the environment a species use would affect the colonization by different pathogen microorganisms (e.g., bacterial, viral). 

Vultures are obligate scavenging birds, which consume a diet based on dead animals [14]. This diet, including tissues in different degrees of decomposition, expose them to diverse pathogenic microorganisms [15]. However, vultures have developed important physiological adaptations to cope with microorganisms present in their diet, such as a stable intestinal microbiome, which outcompete with pathogens ingested [16]. In addition, they possess a low stomach pH that influences the viability of microorganisms ingested and some other particularities that strengthen their immune function (e.g., enhanced innate immunity) [17,18,19]. In the past few years, there was an increase in scientific information showing that vultures can be colonized by diverse pathogen microorganisms, most of them zoonotic and even resistant to antibiotics. This represents a possible threat for them, and a concern regarding the potential influence on public health [20,21,22,23]. Even though there is no current evidence that pathogen spillover may occur from these birds [15], considering its potential relevance it deserves permanent evaluation and further research. Unfortunately, the comparison of pathogenic microorganisms colonizing vultures from different environments with a different degree of anthropic impacts has received little attention (but see, [12,13]). 

In this article, we evaluated if the characteristics of the environments used by vultures could influence the occurrence of specific zoonotic pathogens in these birds. For this, we studied the aerobic bacteria present in vultures, but particularly selected as a target genus *Salmonella* spp.; this is a zoonotic pathogen that is commonly present in birds [10,13,20,21]. The presence of this bacteria was studied in Andean condors (*Vultur gryphus*) using natural environments from northwestern Patagonia, where livestock is raised extensively, and human presence is very low. Results obtained in this wild population were compared with the bacteriological records obtained for captive condors under human care (rehabilitation and handreared condors) from a zoological institution (an anthropized environment). We also identified the occurrence of different non-target microorganisms (genera and species when possible) in oropharynx and cloacae samples of wild and captive Andean condors. In this way, we evaluated the presence of other potential zoonotic pathogens and their affinity with different anatomical regions. Condors from northwestern Patagonia were expected to be rarely exposed to the target bacterial genus since they consume a diet mainly based on carcasses of wild ungulates, hares, and extensive livestock production, in areas with low probability to ingest the zoonotic pathogens studied [13]. Captive condors under human care were expected to present higher occurrence of *Salmonella* spp. compared with the wild ones, since they consume a non-natural diet and live in close contact with humans and other animal species that could be carriers of pathogenic bacteria [24,25,26].

Finally, the results obtained in wild and captive condors were compared with the ones reported for other vulture species from different geographical areas using both anthropized (e.g., rubbish dumps or intensive livestock exploitations) and natural environments, in order to understand the influence of the environment on zoonotic pathogens occurrence. We expected that vulture species using natural environments will present lower occurrences of zoonotic *Salmonella* spp. compared with the ones using anthropized environments, such as sites with intensive livestock practices or rubbish dumps, which are known to be associated with colonization by zoonotic bacteria [13,21]. 

## 2. Materials and Methods

### 2.1. Study Species

The Andean condor is the largest New World vulture (weight up to almost 16 kg, wingspan 3 m) [27,28]. This species inhabits throughout the Andes Mountains, from the north of Venezuela and Colombia to the south of Argentina and Chile [28,29]. It is listed in CITES Appendix I, classified worldwide as Vulnerable by the IUCN red list [30]. The Andean condor population studied from northwestern Patagonia is one of the largest for the species distribution range [31]. In this study area, they feed on large carcasses of sheep, red deer, and cows, but also a large proportion of lagomorphs [32,33]. They forage particularly in the Patagonian steppe, avoiding urbanized sites such as roads, slaughterhouses, and rubbish dumps [13,34,35]. This species is facing different threats associated with human activities in Argentina and throughout their entire distribution range [29,36,37]. For instance, intentional poisoning, lead contamination, and collision with power lines, among others, are common threats present in Argentina, producing injuries in some individuals, which have to be admitted for rehabilitation at specialized rehabilitation centers, such as Buenos Aires Zoo [36,37,38]. 

### 2.2. Study Area

#### 2.2.1. Wild Condors

Wild condors were studied in northwestern Argentine Patagonia, Rio Negro province (41° S and 71° W) (see Supplementary Material Figure S1). This area is a steppe dominated by grasses and shrubs, and it borders the Andean Forest in the west [39]. There are livestock exploitations dedicated to extensive sheep and cattle raising [40,41]. In addition, there are high numbers of introduced mammals, such as red deer (*Cervus elaphus*), wild boars (*Sus scrofa*), and European hares (*Lepus europaeus*) [32,33]. This area has a low degree of human impact. In fact, an important part of this geographical area encompasses land under protection (national parks, provincial reserves, and biosphere reserves) [34]. Moreover, condors in this area travel large distances each day (e.g., 350 km in one day) covering vast natural areas [42].

#### 2.2.2. Captive Condors

The study of captive populations of condors was performed at the Buenos Aires Zoo, in Buenos Aires City (34° S and 58° W), Argentina (see Supplementary Material Figure S2). This zoo was located in an urban environment and received condors suffering from different health problems for rehabilitation. Upon admission, individuals were housed in quarantine enclosures and consumed a diet based in cow fresh meat and lab rodents [43]. In addition, at the Buenos Aires Zoo there was a condor captive rearing program as part of an ex-situ conservation program [44]. Hand reared condors were located in different enclosures, sometimes with other immature individuals to conform pre-release groups, but received the same diet and treatment.

This institution also has a stable collection of diverse mammal, bird, and reptile species. As part of its preventive veterinary program, this zoo routinely performs health studies on captive condors, including bacteriological studies. Both rehabilitation and hand reared condor medical records were consulted for this study.

### 2.3. Sample Design

Wild Andean condors were trapped using cannon nets from 2013 to 2018 in a wild area in North-Western Argentine Patagonia (ca. 41° S 71°W). Each individual trapped was physically evaluated by wildlife veterinarians under physical restraint. Data from captive condors were obtained from medical records dated from 2009 to 2015. All captive individuals were sampled at the Buenos Aires Zoo Veterinary Hospital, under physical restraint. 

Wild and captive individuals were carefully handled to avoid injury and relieve stress following specific approved protocols (Comité Institucional de Cuidado y Uso de Animales de Laboratorio -CICUAL- Facultad de Ciencias Veterinarias-Universidad de Buenos Aires, Resol. 2013/44). Sterile swabs were used to collect separate samples from the oropharynx and cloacae and stored in Stuart transport medium under refrigeration conditions (4 °C) until bacteriological studies. All samples were processed at “Laboratorio Escuela de Enfermedades Infecciosas (LEEI), Facultad de Ciencias Veterinarias”, Universidad de Buenos Aires. The wild population studied included samples of 56 individuals trapped in the surroundings of the city of San Carlos de Bariloche (Rio Negro province, northwest Patagonia, Argentina). The captive group included 71 individuals, 56 condors received for rehabilitation, and 15 hand reared individuals.

### 2.4. Bacteriological Analysis

Bacteriological analyses were performed in order to identify aerobic and facultative anaerobic bacteria, including the detection of *Salmonella* spp., but also considering other non-target potential zoonotic species. The oropharynx samples were cultured on tryptone soy agar (TSA) and blood agar plates (BA) and incubated for 24–48 h at 37 °C. Cloacal swabs were cultured on TSA and xylose lysine deoxycholate (XLD) agar plates at 37 °C for 24 h. Different macroscopic colonies were picked up and culture on TSA to obtain pure cultures and, then, isolates were identified by biochemical tests according to Cowan and Steels (2006) [45]. Cloacal samples were also inoculated into lactose broth at 37 °C for 24 h and after that, inoculum was mixed and a loopful was streaked on XLD and incubated at 37 °C for 24 h. Presumptive *Salmonella* colonies, lactose negative and H_2_S positive, were re-cultured into TSA at 37 °C for 24 h for their identification by biochemical tests. In the case of multiple colonies growth without predominance of any, we consider this sample as polymicrobial. 

*Staphylococcus* spp. was recognized because it produces yellow colonies and the yellow color spreads over the culture medium as the acid produced by the bacteria diffuses. Bacteria with growth and turning yellow in the medium and positive coagulase test were considered *S. aureus*. Antibiotic susceptibility profiles were performed only for *S. aureus* isolates obtained from rehabilitating condors by disk-diffusion method [46], in order to find methicillin resistant S. aureus (MRSA).

### 2.5. Comparison between Condors and Other Vulture Species

In order to compare the results obtained in wild and captive Andean condors with other free ranging vulture species, only cloacae or fecal samples were considered; this is because they are the most common samples collected in studies to diagnose bacteria present in vultures [15]. In addition, *Salmonella* spp. was selected for the comparison because it is a common zoonotic pathogen reported in vultures, and this bacteria can present a threat for other species including humans [15]. To do this, we selected bacteriological studies reporting *Salmonella* spp. on fecal samples or cloacae swabs performed on different vulture species around the world using natural or modified (anthropized) environments. We used the database included in Plaza et al. [15], which comprises a comprehensive compilation of the diverse microorganisms (bacteria, virus, and fungi) present in vultures up to 2020. We then incorporated recently published articles (between the years 2020 and February 2021) studying bacteria in vultures by repeating the same searches done in Plaza et al. [15]. Based on the descriptions presented in each study addressed (Table S1), we classified the environment as anthropized or natural environments. Anthropized sites included studies performed on vultures feeding on rubbish dumps or intensive livestock productions (including supplementary feeding stations provisioned with carcasses from intensive livestock productions). Natural environments included studies performed on vultures feeding on sites of extensive livestock production or pristine geographical areas (including lands under protection). In order to select articles that could be compared with our data, we excluded articles: (1) not reporting number of individuals positive or tested, (2) designed to find novel bacteria strains different than *Salmonella* spp., (3) using methodologies different than traditional bacteriology, (4) searching just for a particular bacteria and not reporting other bacteria isolated, and (5) articles performed in individuals where their origin is not clear or where there is no clear mention about environments where samples were collected (e.g., studies performed with individuals from different origins without specific descriptions). Based on the selection criteria implemented we were able to compare our results in wild and captive condors with 11 articles from 6 countries encompassing 5 vulture species (Table S1). Studies on European species were done in individuals exposed to high anthropization (e.g., intensive farm productions and supplementary feeding stations, Table S1). Studies on American species were performed in a gradient from anthropized environments to wild areas. The wildest areas were the ones where we studied wild Andean condors in Patagonia.

### 2.6. Statistical Analysis

We evaluated the occurrence of *Salmonella* spp. and other non-target potential zoonotic species (e.g., *Escherichia coli*, *Staphylococcus aureus*) isolated in Andean condors, by calculating the number of individuals positive to a specific bacteria over the total individuals sampled x 100. Occurrence of these bacteria was discriminated according to the category of Andean condors studied (wild or captive) and according to the anatomical regions sampled (oropharynx-cloacae). Epitools (https://epitools.ausvet.com.au/, accessed on February 2021) was used to estimate the 95%confidence intervals of *Salmonella* spp. occurrence) using the methodology of Wilson, which is accurate for both large and small numbers of samples [47]. 

To evaluate if the environment where different vultures are studied (anthropized-natural) influences *Salmonella* spp. colonization, the occurrence of this bacteria was computed using the above mentioned methodology; first for each vulture species and then pooling species according to geographical area (America and Europe), but discriminating vultures according to the environment they used when this information was available (Table S1). Finally, a graphical comparison of these occurrences was performed discriminating by species studied, geographical area, and environmental characteristics. For certain species and geographical areas (e.g., Europe) the results only consider birds using highly anthropized environments. 

## 3. Results

**Figure 1:**
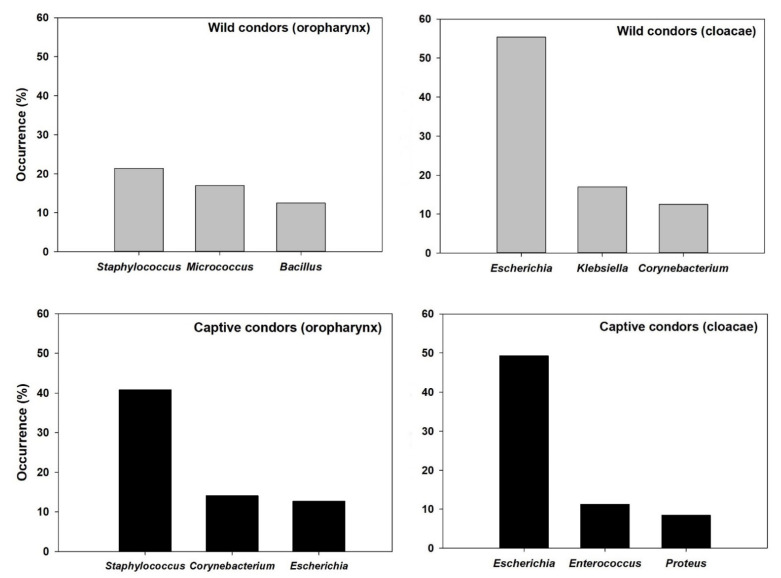
Occurrence of the most representative genus isolated in the oropharynx and cloacae of wild and captive condors.

The diversity of bacteria obtained from the oropharynx of wild individuals was composed by eight genera, being *Staphylococcus*, *Micrococcus,* and *Bacillus* the most frequent genus (Table 1, Figure 1). All samples obtained from cloaca of wild condors were negative for *Salmonella* spp. The diversity of bacteria obtained from cloacae samples of wild individuals was composed by nine genera, being *Escherichia* (represented by *Escherichia coli*) *Klebsiella,* and *Corynebacterium* the most frequent genus (Table 1, Figure 1).

In captive animals, under human care, *Salmonella* spp. was isolated from two of the 71 samples from cloacae (2.8 %). The diversity of bacteria obtained from oropharynx samples was composed by 11 genera and the most frequent of them were *Staphylococcus*, *Corynebacterium,* and *Escherichia* (represented by *Escherichia coli*) (Table 2, Figure 1). The diversity of bacteria obtained from cloacae was composed by nine genera and the most frequent of them were *Escherichia* (represented by *Escherichia coli*), *Enterococcus,* and *Proteus* (Table 2, Figure 1). One *Staphylococcus aureus* isolation from oropharynx of a rehabilitation individual was identified as MRSA. 

**Figure 2:**
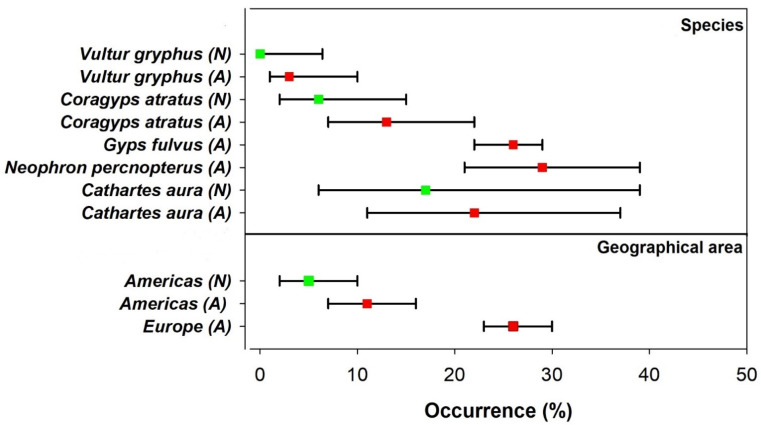
Occurrences (±95% CI) of *Salmonella* spp. according to the environment where different vulture species were sampled indicated as natural (*N*) or anthropized (*A*). The upper part of this figure presents occurrences according to species. Large 95% CI are mainly a result of small sample sizes of the study on a species (Table S1). The lower part of the figure presents occurrences according to the environment and geographical areas (pooling the species). Occurrences for *N. percnopterus* and *G. fulvus* (European species) are shown separately from American species (*V. gryphus, C. aura* and *C. atratus*) because the first two species have only been studied in highly anthropized environments (Table S1).

**Figure 3:**
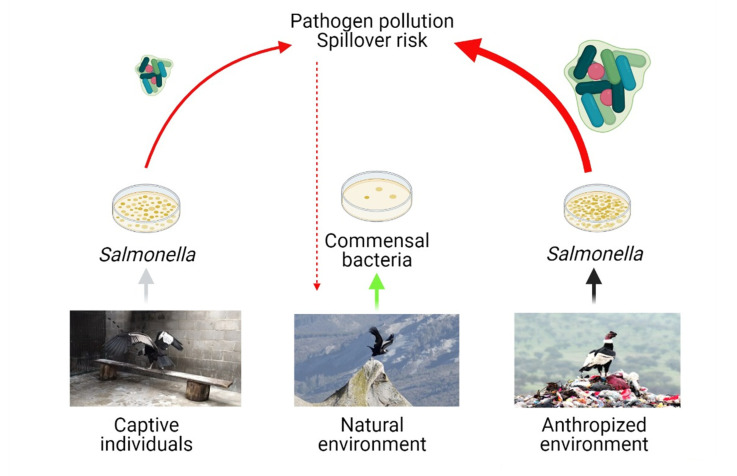
Scheme of the potential link between vultures and ecosystem health, considering vultures using different anthropized environments are more prone to be colonized by zoonotic pathogens than vultures using natural environments. Photographic credits: Captive individuals, Guillermo Wiemeyer; Natural environments, Bruno Osorio; Anthropized environments, Alejandro Olivares.

Finally, our comparison between vulture species suggested that the environment where vultures were sampled might influence the occurrence of *Salmonella* spp. from fecal or cloacae samples, but results are not conclusive (Figure 2 and Figure 3). Vultures of different species and from different geographical areas using anthropized environments showed a tendency to have higher occurrence of *Salmonella* spp. In fact, when pooling all studied vulture species according to the environment used (vultures using anthropized environments vs vultures using natural environments) and geographical areas (Europe vs America) this tendency resulted more notorious, but probably influenced by the large number of studies performed in highly anthropized areas from Europe (Figure 2). 

## 4. Discussion

Wild Andean condors using natural environments in northwest Patagonia were not colonized by *Salmonella* spp. or by any other relevant zoonotic pathogen potentially dangerous for humans. However, we found that some captive condors under human care were positive for *Salmonella* spp. and Methicillin-resistant *S. aureus* (MRSA). Our results suggest that the environment used by condors (where they were sampled) might influence the occurrence of zoonotic pathogens (Figure 3). Based on cloacae samples or fecal samples, vultures form different geographical areas using anthropized environments such as rubbish dumps or intensive livestock sites tend to have higher occurrence of *Salmonella* spp. than vultures sampled in less anthropized environments. However, this merits further research because only a small number of vultures have been studied in both environments and the tendency found can be masked by the low number of samples and by the natural history of the species (e.g., large home ranges, migratory behavior, etc.). Total occurrence of *Salmonella* spp. tend to be higher for vultures from anthropized areas, but this is could be influenced by the large number of studies performed in anthropized areas, mainly represented by European species sampled in territories close to intensive farming and supplementary feeding stations. In this sense, vultures from a highly anthropized geographical area, such as the one sampled in Europe, showed higher occurrences of *Salmonella* spp. compared with vultures from a less anthropized areas in America. Moreover, among American vultures there is a larger occurrence of this pathogen in birds that commonly use anthropized sites such as rubbish dumps (black vultures, *Coragyps atratus* and turkey vultures, *Cathartes aura*) [13]. This suggests the importance of maintaining pristine ecosystems as a precautionary principle. Close contact between humanized areas, intensive livestock practices, and wildlife (wildlife–livestock interface), may promote vulture colonization by dangerous pathogens that may affect their health and increase the possibility of pathogen emergence or spillover from these species. 

Different vulture species around the world have been colonized by *Salmonella* spp. and *Campylobacter jejuni*, especially those species feeding on carcasses provided by intensive farming or using rubbish dumps as food source [13,20,21,48]. In fact, *Salmonella enterica* ser. Typhimurium and ser. Choleraesuis among other serovars, and *Campylobacter jejuni* have been reported colonizing griffon (*Gyps fulvus)* and Egyptian (*Neophron percnopterus*) vultures feeding on pig carcasses provided by supplementary feeding stations (vulture restaurants) in Spain [20,21,48,49]. In fact, most of these bacteria showed antibiotic resistance to common antibiotics used in farming practices (e.g., aminopenicillins) [20]. Similarly, black vultures feeding on rubbish dumps have been reported colonized by different *Salmonella* spp. some of them (e.g., *Salmonella enterica* ser. Typhi, *Salmonella enterica* ser. Paratyphi A) very dangerous for human health [13,50]. On the contrary, our results showed that Andean condors using a less anthropized natural environment in northwestern Patagonia are not colonized by *Salmonella* spp. nor by other relevant zoonotic pathogens. In this sense, our findings add evidence and agree with previous studies reporting an association between pathogen microorganism colonization and characteristics of the environment used by birds [9,10].

In contrast to wild Andean condors, captive condors under human care (rehabilitation or captive reared birds), which are in contact with humans and other captive animals, were colonized by two relevant pathogens *Salmonella* spp. and MRSA. *Salmonella* spp. produces disease in birds characterized by gastroenteritis, fever, sepsis, and even death [51], but it can also affect humans, constituting one of the most prevalent zoonotic diseases in the world [26,52]. Methicillin-resistant *S. aureus* (MRSA) is associated with a variety of conditions, ranging from asymptomatic colonization of the nasal mucosa to mild skin and soft tissue infections and even fulminant invasive disease with high mortality [53]. The findings on captive individuals colonized by those bacteria merit future studies evaluating the influence of human contact and artificial environments in pathogen colonization of these birds. Even when zoological institutions provide controlled safe diets on birds under human care, it is reasonable to assume that the coexistence of non-natural dietary sources, different species cohabiting and variable level of stress could influence the natural (wild) balance of commensal microbiota in condors gut [54]. This situation should be considered for condors and other vultures admitted for rehabilitation, in which different health alterations (infectious, traumatic, etc.) could be complicated with pathogen microorganism colonization. 

It is interesting to note that bacterial microbiota composition changed according to the group of Andean condors evaluated (wild-captive), but also according to the anatomical site of sampling (Figure 1). For instance, *Enterococcus faecalis* and *Enterococcus cloacae* colonized the gastrointestinal tract of captive but not wild condors. These bacteria could be considered as vultures normal microbiota with a positive effect on them, because they produce antimicrobial peptides, useful to cope with microorganism ingested associated with their food [55]. However, these microorganisms have the potential to produce severe health alterations in humans such as endocarditis, urinary tract infections, meningitis, among others [56]. Moreover, *Enterococcus* spp. may potentially resist antibiotics such as vancomycin [55,57]. When comparing the isolates obtained for wild condors according to the anatomical sites examined (cloaca and oropharynx), 6 from 10 identified genera (60%) resulted indistinctly located in any of the sites examined. This might suggest a moderate specificity of association between the identified genera and the anatomical sites. In condors from rehabilitation the situation was similar, since 7 from 13 isolated genera (53.8%) were identified in both locations (oropharynx and cloacae). These values are similar to, but slightly higher than, those reported for griffon vultures where both locations share 47.8% of the genera [58]. The differences we observed in the bacteria present for each group of condors and between anatomical regions could obey multiple factors, which affect the bacterial microbiome, such as characteristics of diet, health conditions, stress levels, and environmental characteristics, among others [59,60]. Further research is needed to evaluate changes in the microbiota produced by different environmental conditions and their potential consequences for vultures but also human health, because some bacteria isolated could produce severe disease in both.

Birds colonized by diverse zoonotic microorganisms have been considered a potential risk to human but also animal health [10]. There is no clear evidence up to date suggesting that vultures play an epidemiological role transmitting pathogens or antibiotic resistance to humans and other species [15]. However, it is reasonable to assume the pathogen spillover might happen under the current particular scenario of global change and ecosystem alteration [1]. Moreover, some pathogenic microorganisms could produce disease in vultures producing a wide range of clinical signs and even the death of some individuals [61,62,63]. This could be of special relevance when these birds live and use anthropized sites, have alterations of their fitness, or are under stress conditions [15]. For instance, wild Griffon, Egyptian and Cinereous (*Aegypius monachus*) vultures have been reported affected by opportunistic mycotic agents probably associated with the consumption of antibiotic residues from carcasses provided by intensive farming [15,63]. Finally, it is key to evaluate microorganism composition in captive individuals considering that in several cases those birds can be reintroduced into natural environments carrying or spreading pathogens to healthy environments and wild individuals.

It is important to note that we did not study strict anaerobic bacteria, however, our findings are relevant since this is the first study addressing and comparing aerobic bacteria from oropharynx and cloaca in a large number of wild and captive Andean condors (but see [64] for *Escherichia coli*). Our study should serve as a starting point for further investigations that deepen and complement the knowledge of the microbiota of vultures using different environments. Moreover, our results are relevant considering the current situation of the threatened Andean condor. This species has populations exploiting anthropogenic food subsidies from rubbish dumps in central Chile, but also Argentina, which could produce diverse health alterations, particularly colonization by dangerous pathogens, which in turn could affect population health [29,65]. 

## 5. Conclusions 

Our results show a low occurrence of zoonotic pathogens colonizing Andean condors from natural areas, with a higher potential risk when they are in human modified environments, such as rehabilitation centers or permanent captivity. In addition, we found that vultures using anthropized environments, such as intensive farming or rubbish dumps, may likely be more colonized by zoonotic pathogens than vultures using natural environments. However, further research is needed to address this issue because studies performed in anthropized versus natural environments including a large number of birds are lacking. Maintaining safe food sources in natural environments could be key to mitigate colonization by zoonotic pathogens in vultures and to prevent potential events of zoonotic pathogen spillover from these species. Considering the current vulture extinction crisis, preventive measures should be taken to minimize anthropogenic impact on vulture species. 

In a scenario of continuous environmental modification, emerging pathogens that originated from wildlife are increasing, with diverse consequences for wildlife but also for human health [1]. One of the most important factors associated with this emergence is related to ecosystem alterations produced by human activities (e.g., changes in the use of the land, presence of rubbish dumps) [1,6]. Moreover, the interface wildlife–livestock and humans could be playing an important epidemiological role in the emergence of novel diseases [1]. Therefore, professionals and technicians involved in manipulation of wild vultures for different purposes, like research, rehabilitation, or conservation, are encouraged to attend safety protocols and wear personal protective equipment in order to minimize both human´s and wildlife health risks. If we continue altering the ecosystem, it is reasonable to assume that the emergence of pathogens will increase with the consequent impact on the species affected and the potential emergence of pathogen microorganisms affecting humans and wildlife, as we have learnt with the pandemic event produced by Covid-19 disease.

## Figures and Tables

**Table 1:** Isolates, occurrence and its 95% confidence interval, of aerobic bacteria isolated from the oropharynx and cloaca in 56 wild Andean condors (Río Negro province, Argentina).

Genus and Species	Oropharynx			Cloacae		
	Isolates	Occurrence	95% CI	Isolates	Occurrence	95% CI
Polymicrobial	26	46.4%	34–59	12	21.43%	13–34
*Corynebacterium* spp.	6	10.71%	5–21	7	12.5%	6–24
*Escherichia coli*	1	1.78%	0–9	31	55,36%	42–64
*Klebsiella* spp.	0	0%	0–6	9	16.97%	9–28
*Proteus mirabilis*	2	3.57%	1–12	2	3.57%	1–12
*Proteus* spp.	0	0%	0–6	3	5.36%	2–15
*Pseudomonas* spp.	1	1,78%	0–9	0	0%	0–6
*Staphylococcus* spp.	8	14.28%	7–26	3	5.36%	2–15
*Staphylococcus aureus*	4	7.14%	3–17	0	0%	0–6
*α-hemolytic Streptococcus*	1	1,78%	0–9	0	0%	0–6
*β-hemolytic Streptococcus*	3	5.36%	2–15	1	1.78%	0–9
*Bacillus* spp.	7	12.5%	6–24	1	1.78%	0–9
*Micrococcus* spp.	9	16.97%	9–28	0	0%	0–6
*Serratia* spp.	0	0%	0–6	2	3.57%	1–12
*Enterococcus* spp.	0	0%	0–6	0	0%	0–6
*Enterobacter* spp.	0	0%	0–6	0	0%	0–6

**Table 2:** Isolates, occurrence and its 95% confidence interval, of aerobic bacteria isolated from oropharynx and cloacae in 71 captive Andean condors (admitted for rehabilitation and hand reared individuals).

Genus and Species	Oropharynx			Cloacae		
	Isolates	Occurrence	95% CI	Isolates	Occurrence	95% CI
Polymicrobial	16	22.53%	14–34	15	21.13%	13–32
*Corynebacterium* spp.	10	14.08%	8–24	1	1.41%	0–8
*Escherichia coli*	9	12.68%	7–22	35	49.30%	38–61
*Klebsiella* spp.	2	2.82%	1–10	0	0%	0–5
*Klebsiella pneumoniae*	1	1.41%	0–8	0	0%	0–5
*Proteus* spp.	0	0%	0–5	6	8.45%	4–17
*Proteus mirabilis*	4	5,63%	2–15	3	4.22%	1–12
*Aeromonas* spp.	1	1.41%	0–8	0	0%	0–5
*Staphylococcus* spp.	21	29.58%	20–41	0	0%	0–5
*Staphylococcus aureus*	8	11.27%	6–21	4	5,63%	2–15
*β-hemolytic Streptococcus*	6	8.45%	4–17	3	4.22%	1–12
*Bacillus* spp.	1	1.41%	0–8	0	0%	0–5
*Micrococcus* spp.	5	7.94%	3–15	1	1.41%	0–8
*Enterobacter* spp.	2	2.82%	1–10	2	2.82%	1–10
*Enterococcus* spp.	0	0%	0–5	7	2.82%	1–10
*Enterococcus faecalis*	0	0%	0–5	4	5,63%	2–15
*Enterococcus cloacae*	0	0%	0–5	2	2.82%	1–10
*Pseudomonas* spp.	2	2.82%	1–10	0	0%	0–5
*Salmonella* spp.	0	0%	0–5	2	2.82%	1–10

## Data Availability

The data that support the findings of this study are available from the corresponding author, upon reasonable request.

## Supplementary Materials

The following are available online at https://www.mdpi.com/article/10.3390/ijerph18105231/s1. Table S1: Studies used to compare our results from wild and captive condors with vulture species using natural or anthropized sites from other parts of the world, Figure S1. Juvenile female wild Andean condor trapped for sampling (Photo: Gonzalo Ignasi) and environment of trapping site in northern Patagonia, Argentina (Photo: Jorgelina Guido), Figure S2. Adult male Andean condor admitted for rehabilitation at Buenos Aires Zoo, Argentina. (Photo: Guillermo Wiemeyer) and Buenos Aires Zoo aerial view (Photographic credit: accessed online https://www.lavoz.com.ar/listas/zoo-de-buenos-aires-supervivientes/, accessed on 13 May 2021).
