# Supplementary material for: Exposure to Anthropogenic Areas May Influence Colonization by Zoonotic Microorganisms in Scavenging Birds
Source: Int J Environ Res Public Health. 2021 May 14;18(10):5231. doi: 10.3390/ijerph18105231 (PMC8156487; doi:10.3390/ijerph18105231)
Supplement: Supplementary file 1 [file ijerph-18-05231-s001.zip › ijerph-1178633-supplementary.pdf]

Supplementary Materials

Table S1. Studies used to compare our results from wild and captive condors with vulture species using natural or anthropized sites from other parts of the world.

Species	Environment*	Occurrence	IC 95%	Country	Reference
Egyptian vulture (*Neophron percnopterus*)	Anthropized	29.2% (28/96)	21.0-38.9	Spain	[1]#
Griffon vulture (*Gyps fulvus*)	Anthropized	61.1% (11/18)	38.6-79.7	Spain	[1]#
Griffon vulture (*Gyps fulvus*)	Anthropized	26.3 % (26/99)	18.6-35.7	Spain	[2]#
Griffon vulture (*Gyps fulvus*)	Anthropized	0% (0/10)	0-27.8	Croatia	[3]#
Griffon vulture (*Gyps fulvus*)	Anthropized	52.6 % (51/97)	42.7-62.2	Spain	[4]
Griffon vulture (*Gyps fulvus*)	Anthropized	21.2 % (22/104)	14.4-30	Spain	[5]
Griffon vulture (*Gyps fulvus*)	Anthropized	13.3 (10/75)	7.4-22.8	Spain	[6]
Griffon vulture (*Gyps fulvus*)	Anthropized	33.3% (5/15)	15.2-58.3	Serbia	[7]#
Griffon vulture (*Gyps fulvus*)	Anthropized	16.7% (31/186)	12-22.7	Spain	[8]**#
Black vulture (*Coragyps atratus*)	Anthropized	33.3 % (1/3)	6.1-79.2	Trinidad	[9]
Black vulture (*Coragyps atratus*)	Anthropized	11.8% (8/68)	6.1-21.5	Argentina	[10]
Black vulture (*Coragyps atratus*)	Natural	6.2% (4/64)	2.5-15.0	Argentina	[10]
Andean condor (*Vultur gryphus*)	Anthropized	2.81 % (2/71)	1.0-9.7	Argentina	This study
Andean condor (*Vultur gryphus*)	Natural	0% (0/56)	0-6.4	Argentina	This study
Turkey vulture (*Cathartes aura*)	Natural	16.7% (3/18)	5.8-39.2	USA	[11]
Turkey vulture (*Cathartes aura*)	Anthropized	21.6% (8/37)	11.4-37.2	USA	[11]
Pooling of vulture species from Europe	Anthropized	26% (184/700)	23-30	Diverse countries	[1-8]
Pooling of vulture species from America	Anthropized	11.0% (19/177)	7-16	Diverse countries	[9-11] and this study
Pooling of vulture species from America	Natural	5.0% (7/138)	2-10	Diverse countries	[9-11] and this study

*** Anthropized refers to vultures feeding in intensive farm productions (e.g., feeding stations) or organic waste from rubbish dumps.**

**** For this study, we only included individuals that were not sampled in previous studies performed by these authors [1, 2].**

# These studies were performed based on fecal samples, while the rest were performed based on cloacae swabs

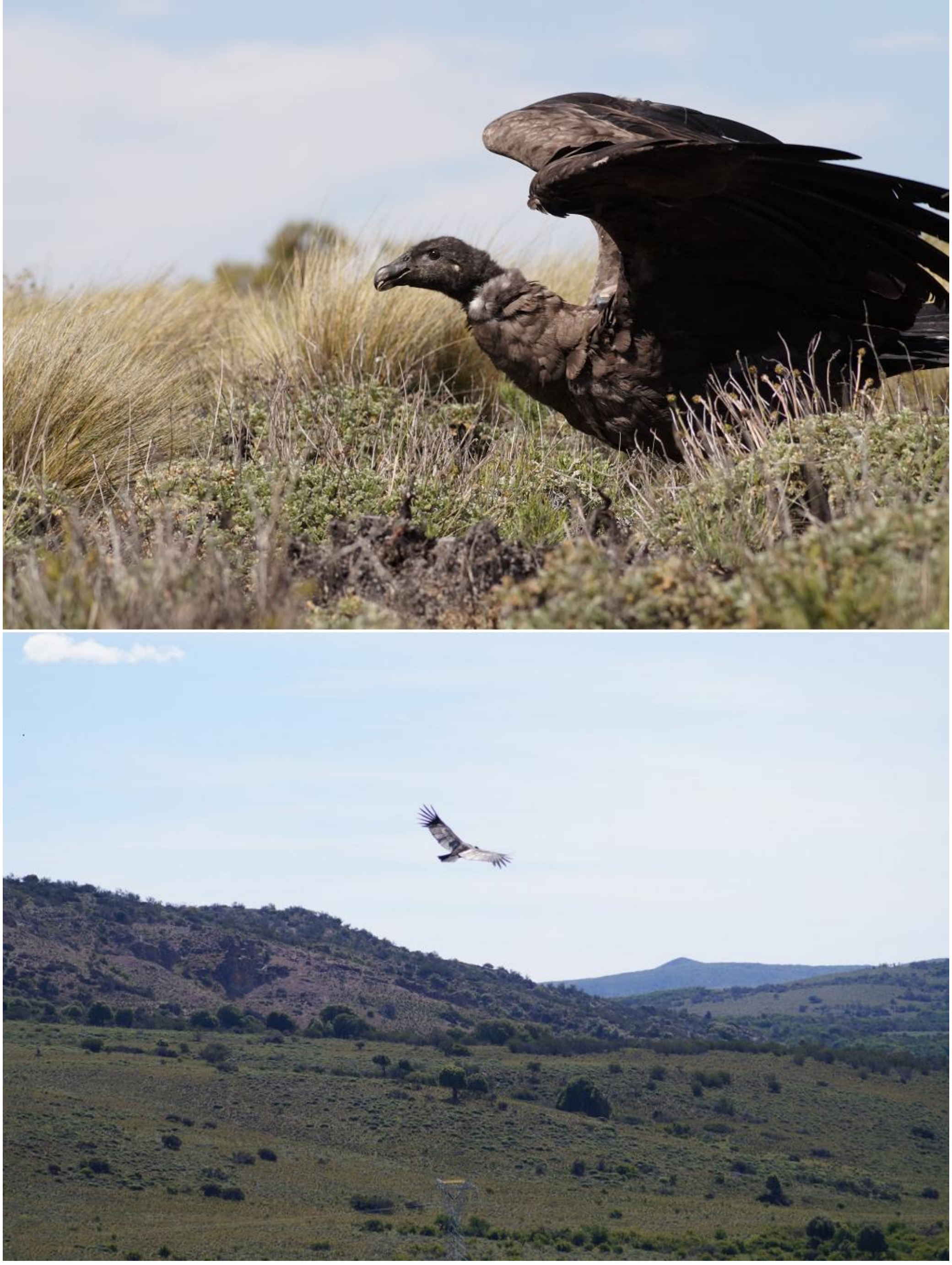

Figure S1. Juvenile female wild Andean condor trapped for sampling (Photo: Gonzalo Ignasi) and environment of trapping site in northern Patagonia, Argentina (Photo: Jorgelina Guido).

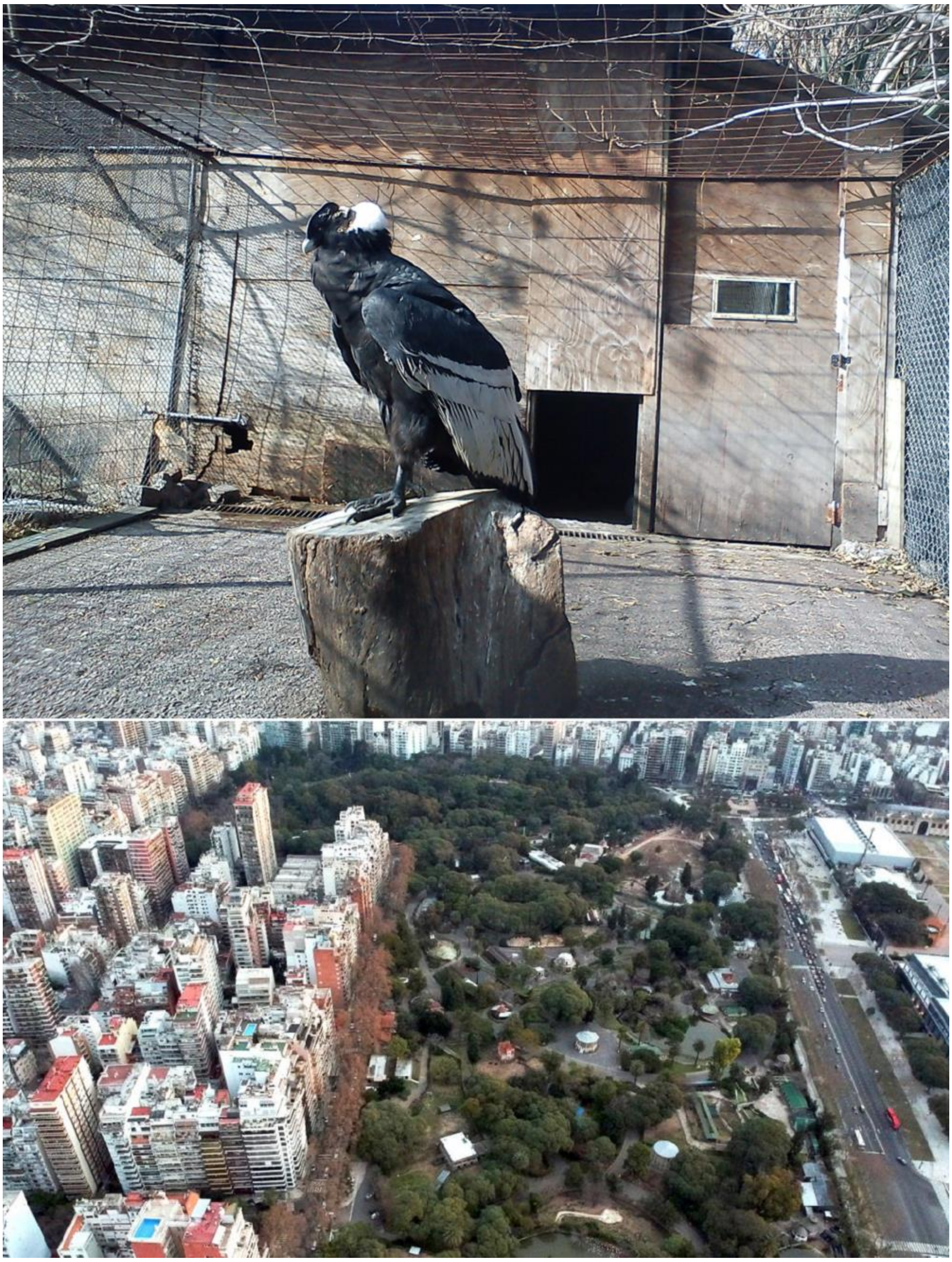

Figure S2. Adult male Andean condor admitted for rehabilitation at Buenos Aires Zoo, Argentina. (Photo: Guillermo Wiemeyer) and Buenos Aires Zoo aerial view (Photographic credit: accessed online https://www.la-voz.com.ar/listas/zoo-de-buenos-aires-supervivientes/)

Supplementary Material References

1. Blanco, G. Supplementary Feeding as a Source of Multiresistant Salmonella in Endangered Egyptian Vultures. *Transboundary and emerging diseases* **2018**.
2. Blanco, G.; de Tuesta, J.A.D. Culture-and Molecular-Based Detection of Swine-Adapted Salmonella Shed by Avian Scavengers. *Science of the Total Environment* **2018**, *634*, 1513–1518.
3. Kocijan, I.; Prukner-Radovčić, E.; Beck, R.; Galov, A.; Marinculić, A.; Sušić, G. Microflora and Internal Parasites of the Digestive Tract of Eurasian Griffon Vultures (Gyps Fulvus) in Croatia. *European Journal of Wildlife Research* **2009**, *55*, 71–74.
4. Marin, C.; Palomeque, M.-D.; Marco-Jiménez, F.; Vega, S. Wild Griffon Vultures (Gyps Fulvus) as a Source of Salmonella and Campylobacter in Eastern Spain. *PLoS One* **2014**, *9*, e94191.
5. Marin, C.; Torres, C.; Marco-Jiménez, F.; Cerdà-Cuéllar, M.; Sevilla, S.; Ayats, T.; Vega, S. Supplementary Feeding Stations for Conservation of Vultures Could Be an Important Source of Monophasic Salmonella Typhimurium 1, 4,[5], 12: I:-. *Science of the Total Environment* **2018**, *636*, 449–455.
6. Vela, A.I.; Casas-Díaz, E.; Fernández-Garayzábal, J.F.; Serrano, E.; Agustí, S.; Porrero, M.C.; Sánchez del Rey, V.; Marco, I.; Lavín, S.; Domínguez, L. Estimation of Cultivable Bacterial Diversity in the Cloacae and Pharynx in Eurasian Griffon Vultures (Gyps Fulvus). *Microb Ecol* **2015**, *69*, 597–607, doi:10.1007/s00248-014-0513-3.
7. Milanov, D.; Fabijan, D.; Prunić, B.; Velhner, M.; Petrović, T. Enteric Bacteria in Fecal Samples of Eurasian Griffon Vultures. *Arhiv Veterinarske Medicine* **2013**, *6*, 45–53.
8. Blanco, G.; de Tuesta, J.A.D. Seasonal and Spatial Occurrence of Zoonotic Salmonella Serotypes in Griffon Vultures at Farmland Environments: Implications in Pathogen Pollution and Ecosystem Services and Disservices. *Science of The Total Environment* **2021**, *758*, 143681.
9. Adesiyun, A.A.; Seepersadsingh, N.; Inder, L.; Caesar, K. Some Bacterial Enteropathogens in Wildlife and Racing Pigeons from Trinidad. *Journal of Wildlife Diseases* **1998**, *34*, 73–80.
10. Plaza, P.I.; Blanco, G.; Madariaga, J.; Boeri, E.; Teijeiro, M.L.; Bianco, G.; Lambertucci, S. Scavenger Birds Exploiting Rubbish Dumps: Pathogens at the Gates. *Transboundary and emerging diseases* **2018**.
11. Sulzner, K.; Kelly, T.; Smith, W.; Johnson, C.K. Enteric Pathogens and Antimicrobial Resistance in Turkey Vultures (Cathartes Aura) Feeding at the Wildlife–Livestock Interface. *Journal of Zoo and Wildlife Medicine* **2014**, *45*, 931–934.
